# RRP8, associated with immune infiltration, is a prospective therapeutic target in hepatocellular carcinoma

**DOI:** 10.1007/s00432-024-05756-9

**Published:** 2024-05-09

**Authors:** Kai You, Xingxing Du, Yunzheng Zhao, Fukai Wen, Zhaoyang Lu, Huitao Fan

**Affiliations:** 1 NHC Key Laboratory of Cell Transplantation, The First Affiliated Hospital of Harbin Medical University, Harbin, Heilongjiang Province China; 2 Key Laboratory of Hepatosplenic Surgery of Ministry of Education, The First Affiliated Hospital of Harbin Medical University, Harbin, Heilongjiang Province China; 3 Department of Critical Care Medicine, The First Affiliated Hospital of Harbin Medical University, Harbin, Heilongjiang Province China

**Keywords:** RRP8, Targeted therapy, Prognosis, Immune infiltration, Hepatocellular carcinoma

## Abstract

**Background:**

Ribosomal RNA Processing 8 (RRP8) is a nucleolar Rossman fold-like methyltransferase that exhibits increased expression in many malignant tumours. However, the role of RRP8 in hepatocellular carcinoma (HCC) is still uncertain. We explored the relationships between RRP8 and prognosis and immune infiltration, as well as the putative pathological function and mechanism of RRP8 in HCC.

**Methods:**

Analysis of RRP8 expression across cancers was performed by using multiple databases. Associations between RRP8 expression and clinicopathological factors were further examined. Gene enrichment analysis was used to identify various putative biological activities and regulatory networks of RRP8 in HCC. The relationship between RRP8 expression and immune infiltration was confirmed by single-sample gene set enrichment analysis (ssGSEA). Univariate and multivariate Cox regression analyses were conducted to assess the impact of clinical variables on patient outcomes. Furthermore, a nomogram was constructed to estimate survival probability based on multivariate Cox regression analysis. Functional validation of RRP8 in HCC was performed with two different systems: doxycycline-inducible shRNA knockdown and CRISPR-Cas9 knockout.

**Results:**

RRP8 was markedly overexpressed in HCC clinical specimens compared to adjacent normal tissues. Further analysis demonstrated that RRP8 was directly connected to multiple clinical characteristics and strongly associated with various immune markers in HCC. Moreover, elevated RRP8 expression indicated an unfavourable prognosis. Our functional studies revealed that both knockdown and knockout of RRP8 dramatically attenuated liver cancer cells to proliferate and migrate. Knockout of RRP8 decreased the phosphorylation of MEK1/2 and β-catenin-(Y654) signalling pathway components; downregulated downstream signalling effectors, including Cyclin D1 and N-cadherin; and upregulated E-cadherin.

**Conclusions:**

RRP8 is strongly implicated in immune infiltration and could be a potential therapeutic target in HCC.

**Supplementary Information:**

The online version contains supplementary material available at 10.1007/s00432-024-05756-9.

## Introduction

Globally, 841,000 new cases of HCC are diagnosed each year, and 782,000 deaths are caused by the disease (Villanueva and Longo 2019). HCC development is influenced by a number of important factors, including hepatitis B infection, hepatitis C infection and exposure to aflatoxin-tainted foods (Howell et al. 2021). HCC is typically treated with surgery; however, the propensity for recurrence and metastasis makes it very challenging to successfully treat (Johnson et al. 2022). Hence, novel and useful biomarkers or targeted therapies are urgently needed to diagnose HCC early and treat it effectively (Bruix et al. 2016; Frau et al. 2010; Gu et al. 2023).

The RRP8 protein is located mainly in the nucleus and harbours highly conserved methyltransferase-specific motifs (Bousquet-Antonelli et al. 2000). It forms an energy-dependent nuclear silencing complex with SIRT1 and SUV39H1, which control ribosomal RNA (rRNA) production when the cellular energy status changes (Murayama et al. 2008). It was reported that RRP8 is highly expressed and is incorporated in the predictive response score (pRS) algorithm for neoadjuvant treatment in breast cancer (Han et al. 2021). A recent study also showed that m6A/m5C/m1A regulates subsets of genes, which include RRP8 (Li et al. 2022), in HCC. However, a comprehensive analysis of the pathological function and potential molecular mechanism in HCC is still lacking.

In this study, we analysed different publicly available datasets and used two different HCC cell models and cancerous tissues from HCC patients to examine RRP8 expression and function. In addition, we evaluated the connection between RRP8 expression and prognostic risk in HCC. We then investigated the probable biological activities of RRP8 in HCC and the associated signalling networks via gene enrichment analysis. Finally, data from our functional knockdown and knockout experiments revealed that RRP8 is essential for liver cancer cells to proliferate and migrate by inhibiting the MEK1/2 and β-catenin signalling pathways. Taken together, our findings elucidate the indispensable functions of RRP8 in HCC and provide a theoretical basis for a targeted therapy for HCC.

## Materials and methods

### TCGA data analysis

We obtained Level 3 RNA-Seq expression profile data for 374 hepatocellular carcinoma tissues and 50 normal tissues from The Cancer Genome Atlas (TCGA) database (https://portal.gdc.cancer.gov/), and the related clinical information was also obtained (Blum et al. 2018). The RNA-Seq data was converted from FPKM to TPM format, then stored and analyzed (Supplementary Table 1).

### Patients and clinical specimens

Thirty-six paired HCC tissues and adjacent normal tissues were obtained from the First Affiliated Hospital of Harbin Medical University between February 2020 and May 2023. The ethics committee of the First Affiliated Hospital of Harbin Medical University approved the study (ethical approval number: 201909).

### Cell culture

WRL68, PLC5, MHCC-97H, HepG2, HCCLM3 and Huh7 cells were used in this study. WRL68 was a normal liver cell line purchased from SAIBAIKANG (Shanghai, China). PLC5, MHCC-97H, and HepG2 cells were obtained from the National Collection of Authenticated Cell Cultures (Shanghai, China), HCCLM3 cells were obtained from EK-Bioscience Biotechnology (Shanghai, China), Huh7 cells were obtained from Procell Life Science & Technology (Wuhan, China), the above five cell lines are liver cancer cell lines. RPMI-1640 medium was utilized for WRL68 cells, MEM was used for PLC5 and HepG2 cell culture, and DMEM was used for culture of the other cell lines. All medium were supplemented with 10% FBS and 1% penicillin/ streptomycin. The cell lines were cultured at 37 °C and 5% CO_2_ for optimal growth.

### Virus packaging and infection

Tet-pLKO-puro (Addgene #21,915) and lentiCRISPRv2-puro vectors (Addgene #98,290) were used to construct the RRP8 shRNA and RRP8 sgRNA lentiviral plasmids, respectively. Plasmid DNA was extracted with a ZR Plasmid Miniprep^™^-Classic kit (Zymo, D4016). HEK-293T cells were transfected with the shRNA, sgRNA packaging (psPAX2) and envelope (pMD2.G) plasmids using PEI (Sigma‒Aldrich, 919,002). The virus-containing supernatants were filtered through a 0.45 μm strainer (Biosharp, catalogue# BS-PES-45). Lentiviruses were packaged and used to infect liver cancer cells to establish cell lines with stable RRP8 knockdown and RRP8 knockout. Cells were screened using 2 μg/ml puromycin (RHAWN, catalogue# R032317) for 7 days before use. The sequences of the targeted RRP8 shRNAs and RRP8 sgRNA oligonucleotides are provided in Supplementary Table 4.

### Quantitative real-time PCR

An EasyPure^®^ RNA Kit (TransGen Biotech, catalogue# ER101-01) was used for RNA extraction. Gene expression was quantified using Hieff UNICON^®^ Universal Blue qPCR SYBR Green Master Mix (Yeasen, catalogue# 11184ES08). The sequences of primers used for RRP8 amplification are shown in Supplementary Table 4.

### Western blotting

The RIPA lysis buffer (Beyotime, catalogue# P0013B) was used to extract total protein from HCC tissues and cell lines. The proteins were then transferred onto nitrocellulose membranes with 300 mA for a duration of 90 min. Following the blocking step with 5% skim milk for a duration of 2 h, the NC membranes (Millipore, catalogue# HATF00010) were subjected to overnight incubation with primary antibodies at a temperature of 4 °C. The primary antibodies against RRP8 (catalogue# 20,129-1-AP, 1:1000) and GAPDH (catalogue# 10,494–1-AP, 1:2000) were obtained from Proteintech. The primary antibodies against N-cadherin (catalogue# 13,116, 1:1000) and E-cadherin (catalogue# 3195, 1:1000) were obtained from Cell Signaling Technology. The primary antibodies against p-β-Catenin-(Y654) (catalogue# sc-57533, 1:600), β-catenin (catalogue# sc-7963, 1:600), p-MEK1/2 (catalogue# sc81503, 1:600), MEK1/2 (catalogue# sc-81504, 1:600), and CyclinD1 (catalogue# sc-8396, 1:1000) were obtained from Santa Cruz Biotechnology. These signals were detected using an enhanced chemiluminescence (ECL) detection kit (Cat. 32,106, Pierce Biotechnology, USA). ImageJ software was used to analyse band intensities.

### Analysis of differentially expressed genes (DEGs)

Patients with HCC were divided into the high and low RRP8 expression groups based on the median RRP8 expression value. The "limma" package was used to perform differential expression analysis between the high and low RRP8 expression groups (Ritchie et al. 2015). DEGs were identified by the following threshold criteria: adjusted *P* < 0.05 and |log2 fold change (FC)|> 1. Volcano plots and heatmaps were used for visualization.

### Functional analysis of DEGs

The "ClusterProfiler" R package (Yu et al. 2012) was employed to perform Kyoto Encyclopedia of Genes and Genomes (KEGG) enrichment analysis (Kanehisa 2000). Gene Ontology (GO) functional enrichment analysis including the biological process (BP), cellular component (CC), and molecular function (MF) categories was also performed with the "ClusterProfiler" R package. Gene set enrichment analysis (GSEA) was employed to perform pathway enrichment analysis (http://www.gsea-msigdb.org/gsea/index.jsp) (Subramanian et al. 2005). An FDR value of 0.05 was used as a cut-off to identify the significantly enriched pathway terms.

### Receiver operating characteristic (ROC) curve analysis

The "pROC" package (Robin et al. 2011) was used to conduct ROC analysis to assess the diagnostic potential of RRP8 in distinguishing HCC tissues from normal tissues. The area under the curve (AUC) value has a potential range between 1.0 (indicating perfect diagnostic value) and 0.5 (indicating no diagnostic value).

### Tumour-associated immune infiltration analysis

We used ssGSEA (Hänzelmann et al. 2013) to evaluate the infiltration of 24 immune cell types by utilizing known immune signatures available in the published literature (Bindea et al. 2013). Genomic variation was analysed using TIMER (https://cistrome.shinyapps.io/timer/) to confirm the correlations between RRP8 and immune cell marker expression according to the methods in the article (Li et al. 2017). The relationship between RRP8 expression and immune infiltration was assessed using the cor.test function in R, while the Wilcoxon rank-sum test was employed to compare levels of immune cell infiltration in groups with high and low RRP8 expression.

### Protein‒protein interaction (PPI) network construction

The STRING database (https://cn.string-db.org/) (Mering et al. 2003; Szklarczyk et al. 2019) was used to construct a PPI network of the coregulated DEGs, which was subsequently visualized with Cytoscape.

### Survival analysis

The log-rank test was employed to assess the differences in survival rates between groups with high and low RRP8 expression levels. Data were presented using a Kaplan–Meier curve. Univariate and multivariate Cox regression analyses were conducted to assess the impact of clinical variables on patient outcomes. The "forestplot" R package was utilized to visually present the *P* value, hazard ratio (HR), and 95% confidence interval (CI) of each variable.

### Construction and validation of a nomogram

A nomogram was constructed through multivariate Cox analysis to forecast the overall survival probability. The performance of the nomogram was evaluated through calibration plots, while the discrimination ability was quantified using the concordance index (C-index). The nomogram and calibration plots were generated utilizing the "RMS" R package (Jeong et al. 2020).

### Interactions of RRP8 with chemicals and genes

The Comparative Toxicogenomics Database (CTD; http://ctdbase.org/) is an online resource that aids in the investigation of novel associations between molecular mechanisms and chemicals that impact health outcomes. In this study, we utilized the CTD to examine the chemicals that interact with RRP8 and identified genes strongly related to RRP8 based on the common interacting chemicals.

### In vitro assays for proliferation and migration

We utilized a Cell Counting Kit-8 (CCK-8) (Beyotime, catalogue# C0043) to evaluate the proliferative capacity of liver cancer cells. The CCK-8 assay was performed with 2000 cells per well in a 96-well plate. The cells were incubated with 100 µl of CCK-8 solution at 37 °C for 2 h. The absorbance of each well after incubation was measured at 450 nm. For the colony formation assay, 2000 cells were seeded in one well of a 6-well plate. Then, after 12–14 days, 4% paraformaldehyde was added to the wells, and the cells were visualized with 0.5% crystal violet (Biosharp, catalogue# BS941). Migration assays were carried out with 8 μm-pore size chamber (Corning, catalogue# 3422); Each well's upper chamber was seeded with 8 × 10^4^ cells in 300 μl of serum-free culture medium, while the lower chamber was filled with 500 μl of medium containing 20% fetal bovine serum. After 48 h, cells were fixed with 4% paraformaldehyde and stained with 0.5% crystal violet for 30 min, then imaged and counted.

### Statistical analysis

In this study, statistical analysis was conducted using R (version 4.2.1). Wilcoxon rank-sum and Wilcoxon signed-rank tests were used to compare unpaired and paired samples, respectively. *P* < 0.05 was considered statistically significant.

## Results

### RRP8 is overexpressed in HCC

**Fig. 1:**
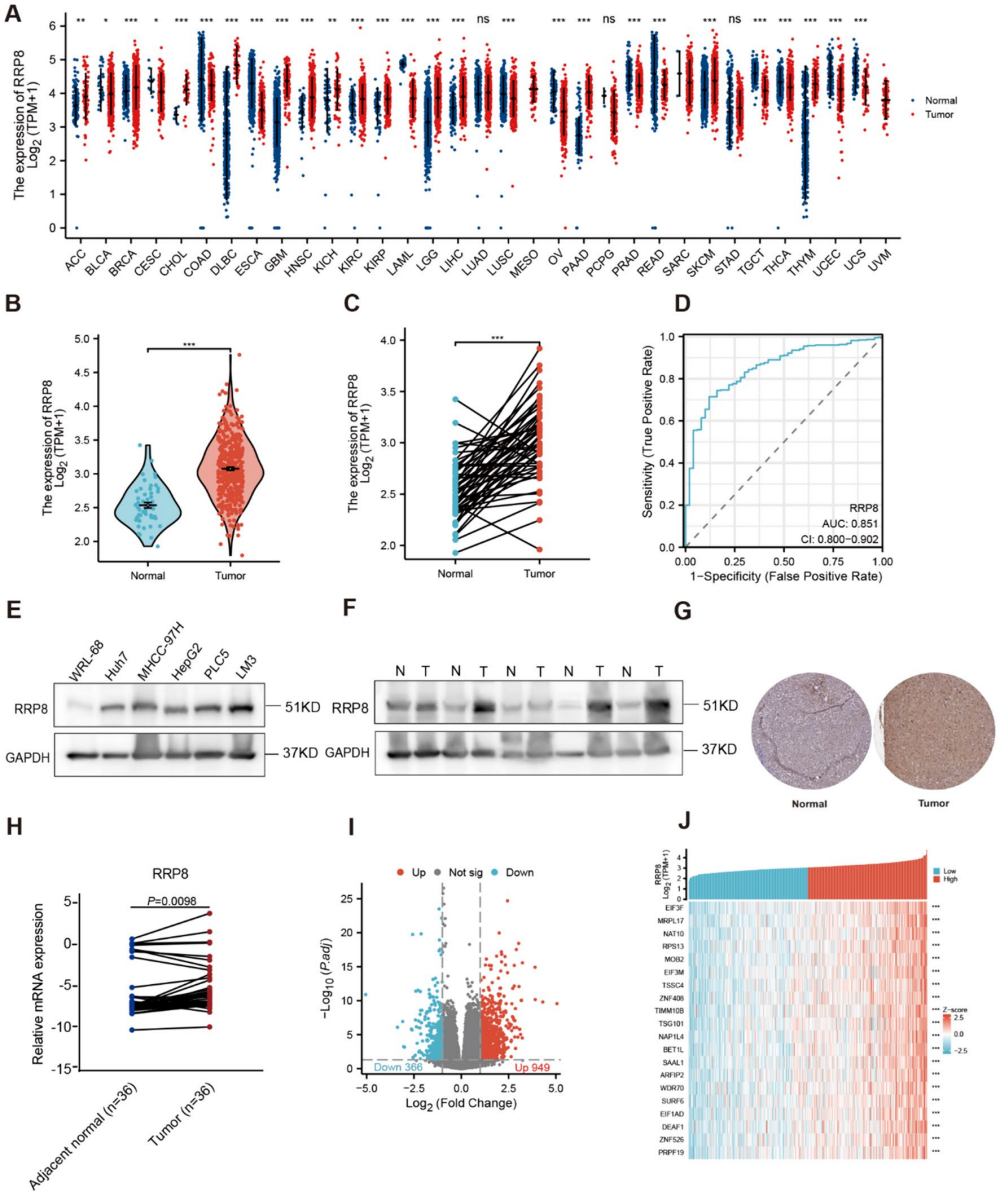
Differential expression of RRP8 in HCC and RRP8-related DEGs analysis. **A** Differential expression of RRP8 in various cancerous specimens in comparison with normal tissues (GTEx and TCGA). **B**, **C** Expression of RRP8 in HCC specimens (TCGA).** D** The diagnostic value of RRP8 in HCC specimens was assessed using ROC curves (TCGA).** E** Protein expression levels of RRP8 were examined in WRL68, Huh7, MHCC-97H, HepG2, PLC5 and LM3 cell lines.** F** Protein expression levels of RRP8 in five pairs of matched adjacent normal and HCC tissues. **G** Protein expression levels of RRP8 in normal tissues and HCC were detected by IHC (HPA). **H** The mRNA expression levels of RRP8 in 36 pairs of adjacent normal and HCC tissues. **I** Volcano plot of DEGs (TCGA).** J** Heat map of 20 genes closely associated with RRP8 (TCGA). *P < 0.05; **P < 0.01; ***P < 0.001; *N* adjacent normal tissue, *T* tumor tissue; ns: not significant

First, we evaluated RRP8 expression in various cancers and normal tissue types using TCGA and Genotype-Tissue Expression database (GTEx) (https://gtexportal.org/home/). RRP8 was found to be overexpressed in 16 types of cancer, including HCC (Fig. 1A). In addition, compared to that in paired normal tissue samples, RRP8 expression was increased in 5 types of cancer (Supplementary Fig. 1). TCGA dataset contained 374 HCC tissues and 50 normal tissues, the expression of RRP8 was significantly elevated in tumor specimens (Fig. 1B). Similar results were obtained by analysis of matched normal and HCC samples (Fig. 1C). ROC curve analysis, a correlation analysis method providing specificity and sensitivity data, revealed an area under the curve (AUC) of 0.851 for RRP8. These findings indicate that RRP8 may be a good diagnostic indicator for differentiating HCC tissue from normal tissue in TCGA dataset (Fig. 1D). The Western blot results demonstrated that RRP8 was highly expressed in Huh7, MHCC-97H, HCCLM3, HepG2, and PLC5 liver cancer cells (Fig. 1E). We then confirmed that RRP8 was overexpressed in the above-described five tumour tissues compared to the matched adjacent normal tissues by Western blotting (Fig. 1F). In addition, immunohistochemistry (IHC) of samples from the Human Protein Atlas (HPA) database (https://www.proteinatlas.org/) further confirmed that RRP8 was markedly overexpressed in tumour specimens compared with normal tissues (Fig. 1G). Furthermore, our qRT-PCR results showed higher RRP8 mRNA expression in 36 HCC tissue samples compared to matched normal tissue samples (Fig. 1H).

### Characterizing the RRP8-related DEGs in HCC

For comparison, we subsequently categorized HCC patients into high and low RRP8 expression groups based on RRP8 expression levels in the TCGA database. The significance threshold criteria were defined as an absolute log2 fold change > 1 and an adjusted *P* value < 0.05. RNA-Seq data analysis revealed a total of 1315 differentially expressed genes (DEGs), 949 of which were associated with high RRP8 expression and 366 of which were correlated with low RRP8 expression (Fig. 1I; Supplementary Table 3). The correlations between the expression of RRP8 and 20 selected genes were visualized in a heatmap (Fig. 1J).

### Functional analysis of RRP8

**Fig. 2:**
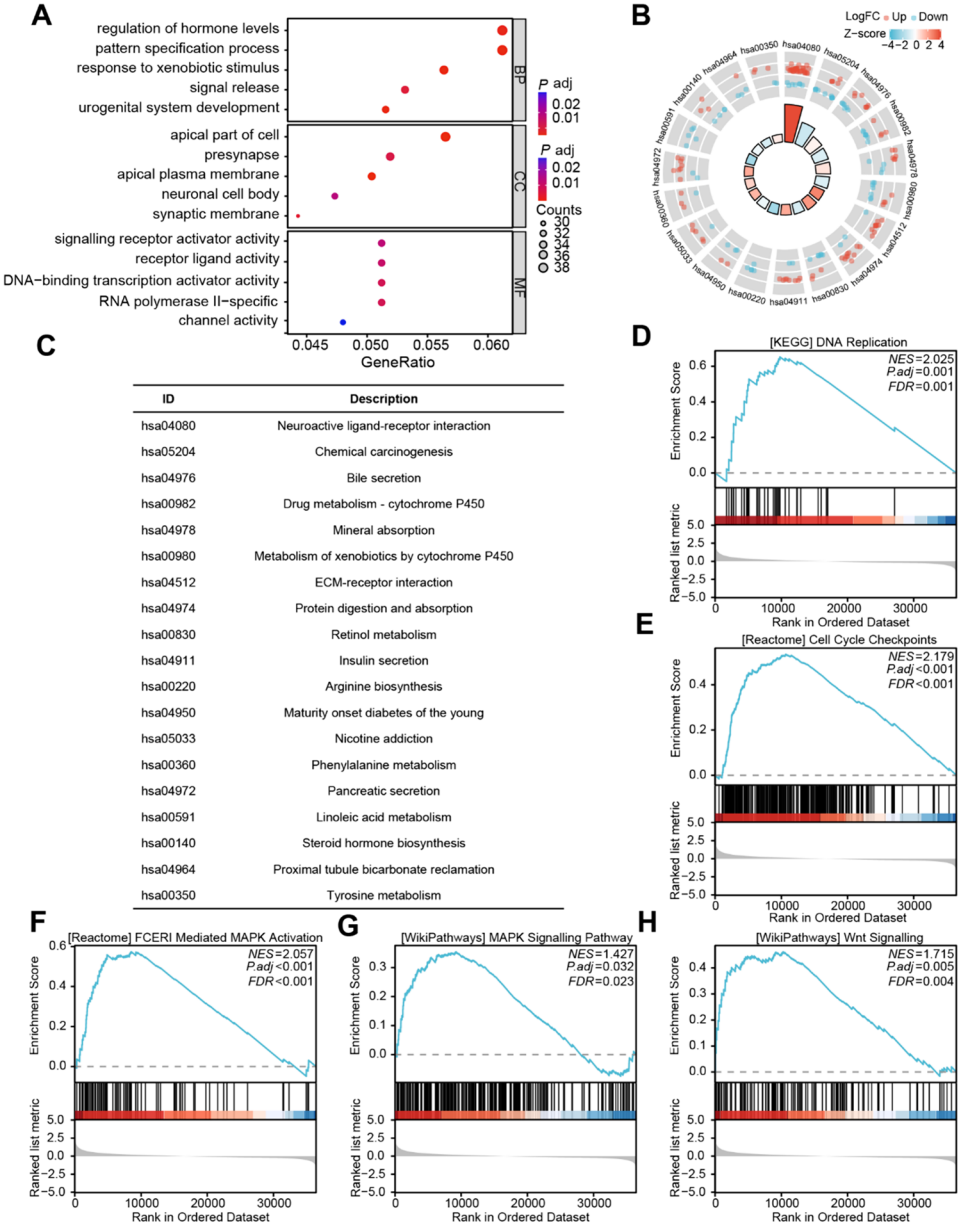
Analysis of the functional enrichment of RRP8-associated genes in HCC. **A** Pathways in GO classifications that were enriched in HCC. **B**, **C** Analysis of KEGG pathways using RRP8-related DEGs. **D–H** GSEA analysis of RRP8-related genes enriched in DNA Replication (**D**), Cell Cycle Checkpoints (**E**), FCERI Mediated MAPK Activation (**F**), MAPK Signalling Pathway (**G**), Wnt Signalling Pathway (**H**). The data comes from the TCGA database. *NES* Normalized Enrichment Score, *FDR* False Discovery Rate

To explore the putative biological activities of the RRP8-related DEGs, the "clusterProfiler" R package was utilized to performed GO and KEGG enrichment analyses. GO analysis indicated that the RRP8-related DEGs were linked to the activity of signalling receptor activators, receptor ligands, DNA-binding transcriptional activators, and channels (Fig. 2A; Supplementary Table 5). The DEGs associated with RRP8 exhibited enrichment in chemical carcinogenesis, retinol metabolic processes, neuroactive ligand‒receptor, ECM–receptor interaction, bile secretion, insulin secretion, and tyrosine metabolism pathways, according to the KEGG analysis results (Fig. 2B, C; Supplementary Table 5). GSEA was utilized to evaluate RRP8-related signalling pathways in HCC. In patients with high RRP8 expression, the subsequent pathways exhibited significant enrichment: DNA Replication, Cell Cycle Checkpoints, FCERI Mediated MAPK Activation, MAPK Signalling Pathway, Wnt Signalling Pathway (Fig. 2D–H; Supplementary Table 6).

### PPI network analysis

**Fig. 3:**
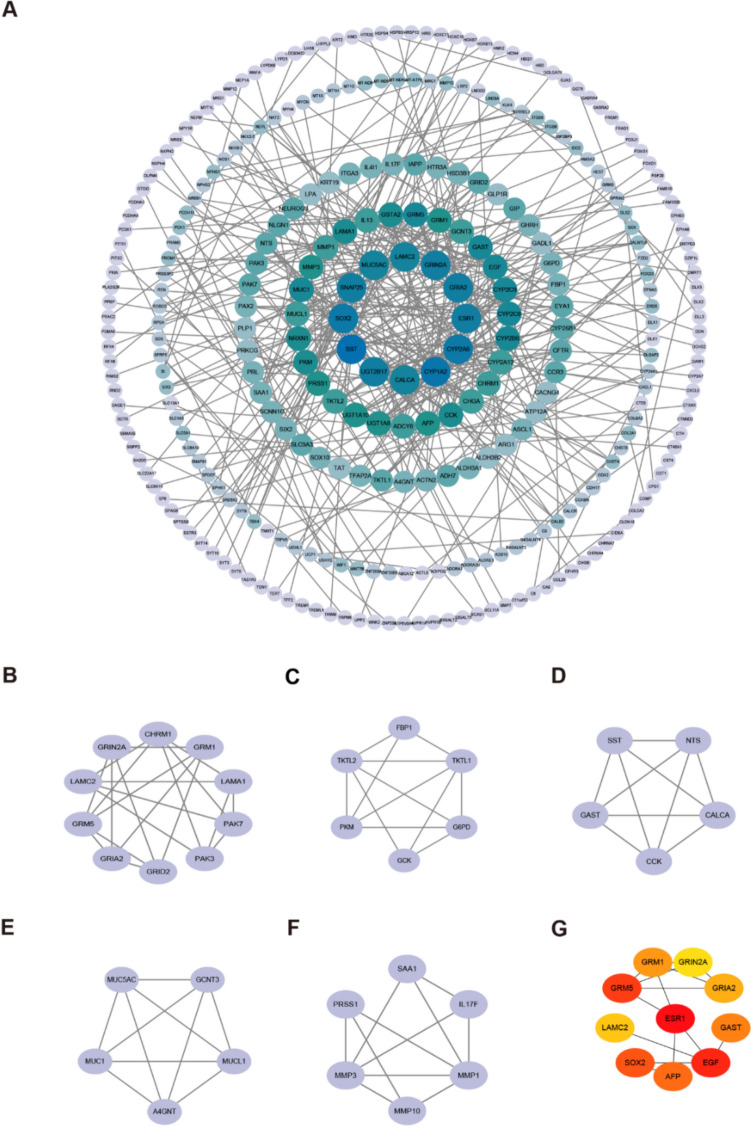
Enrichment analysis of PPI networks**. A** The network was constructed by PPI pairings derived from the STRING dataset. **B–F** Hub clusters of genes selected from the PPI network with the criteria that total score more than 5000.** G** Seven dominant hub genes of the PPI network. The data comes from the TCGA database. *PPI* Protein–Protein Interaction

We next investigated the PPIs among the proteins encoded by the 1315 DEGs by utilizing the STRING database, which was subsequently visualized with Cytoscape. The underlying mechanisms were further explored by adjusting the correlation threshold to 0.70 and constructing the PPI network (Fig. 3A; Supplementary Table 7). After screening of 304 proteins and 427 edges, five key gene clusters with a total score ≥ 5000 were identified (Fig. 3B–F). Moreover, the top ten core genes, namely, ESR1, GRM5, EGF, GAST, AFP, SOX2, LAMC2, GRIA2, GRIN2A and GRM1, were further examined (Fig. 3G).

### Correlation analysis of RRP8 expression and immune cell infiltration

**Fig. 4:**
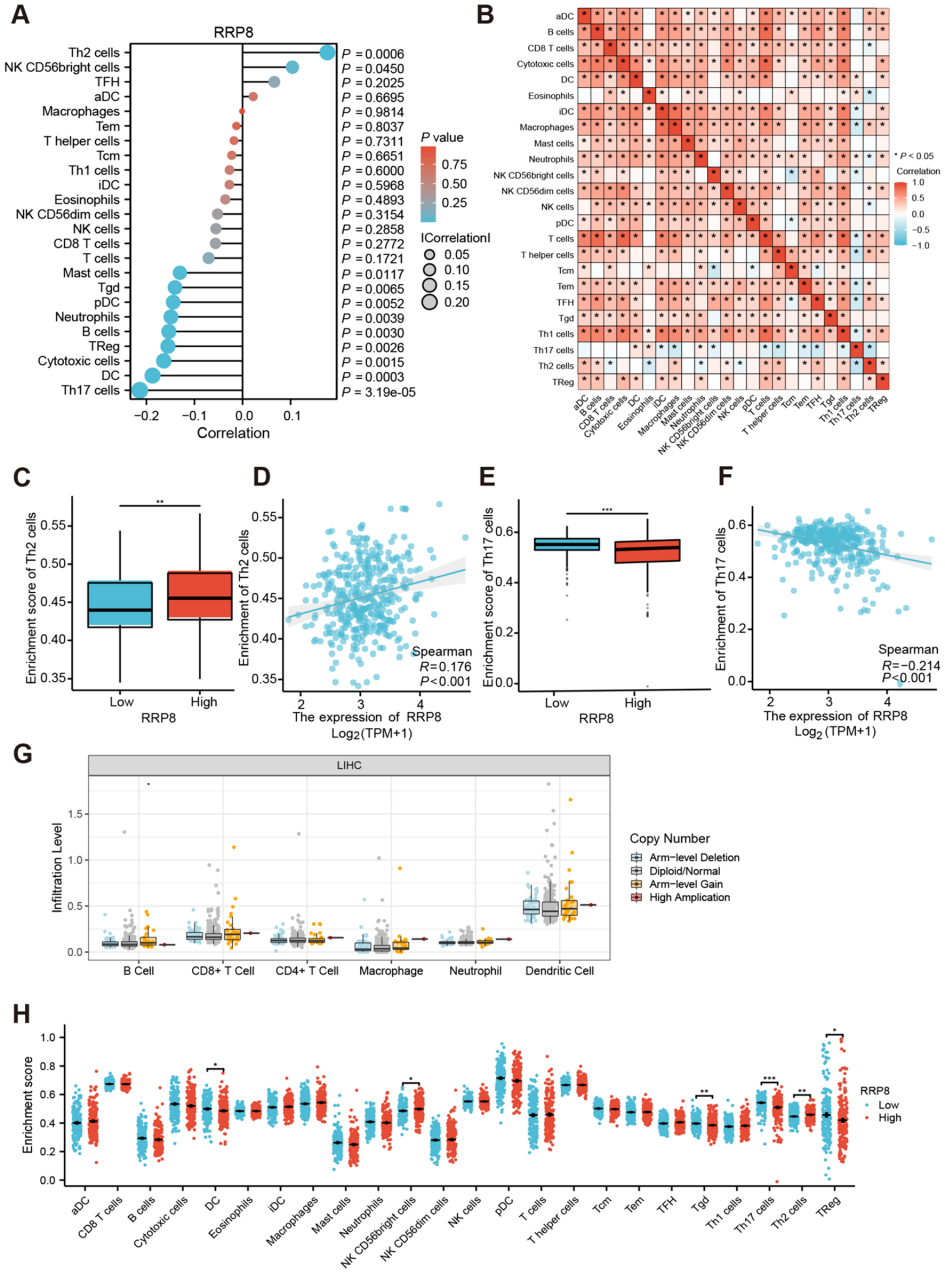
Comprehensive analysis of RRP8 expression in immune infiltration. **A** Correlation between expression of RRP8 and the relative accumulations of 24 immune cell subsets was plotted by ssGSEA (TCGA). **B** Heat map of association of 24 infiltrating immune cells in HCC (TCGA). Scatter diagrams depicting the differences of Th2 cells (**C**, **D**) and Th17 cells (**E**, **F**) between high and low RRP8 expression subgroups (TCGA). **G** SCNA module indicate that no significant association between RRP8 CNV and immune cells in HCC (TIMER). **H** The relationship between expression levels of RRP8 and 24 immune cells (TCGA). *SCNA* Somatic Copy Number Alterations, *CNV* Copy Number Variations

Next, we conducted a Spearman correlation analysis to assess the association between RRP8 expression and the infiltration of immune cells for HCC patients identified by ssGSEA in TCGA database. Infiltration of natural killer (NK) CD56bright cells and T helper 2 (Th2) cells exhibited a positive correlation with RRP8 expression. Infiltration of B cells, neutrophils, T helper 17 (Th17) cells, dendritic cells (DCs), cytotoxic cells, regulatory T cells (TRegs), plasmacytoid dendritic cells (pDCs), gamma delta T (Tgd) cells and mast cells was inversely associated with RRP8 expression (Fig. 4A). By generating a heatmap, we assessed and visualized correlations between the ratios of 24 distinct tumour-infiltrating immune cell subsets (Fig. 4B). The amount of Th2 cell infiltration was positively correlated with the level of RRP8 expression (Fig. 4D) and was notably high in the group with elevated RRP8 expression (Fig. 4C). Th17 cell infiltration was negatively associated with RRP8 expression (Fig. 4F), and Th17 cells were less enriched in high RRP8 expression group (Fig. 4E). These findings demonstrate that RRP8 is crucial for HCC immune infiltration. However, the variation in the somatic copy number alteration (SCNA) module from TIMER database analysis did not reveal a significant association between RRP8 copy number variation (CNV) and immune cell infiltration in HCC (Fig. 4G). Furthermore, HCC specimens were categorized into subgroups with low or high RRP8 expression to determine whether there were variations in the HCC immunological microenvironment (Fig. 4H). The numbers of NK CD56bright and Th2 cells were strongly increased in the group with elevated RRP8 expression, whereas the numbers of DCs, Tgd cells, Th17 cells and TReg cells were decreased. In addition, TIMER database analysis revealed that RRP8 was positively correlated with six critical immune cell types (Supplemental Fig. 2A–G). These findings confirm that RRP8 overexpression is closely correlated with immune cell infiltration in HCC.

### Correlations of RRP8 expression with clinicopathological features

**Fig. 5:**
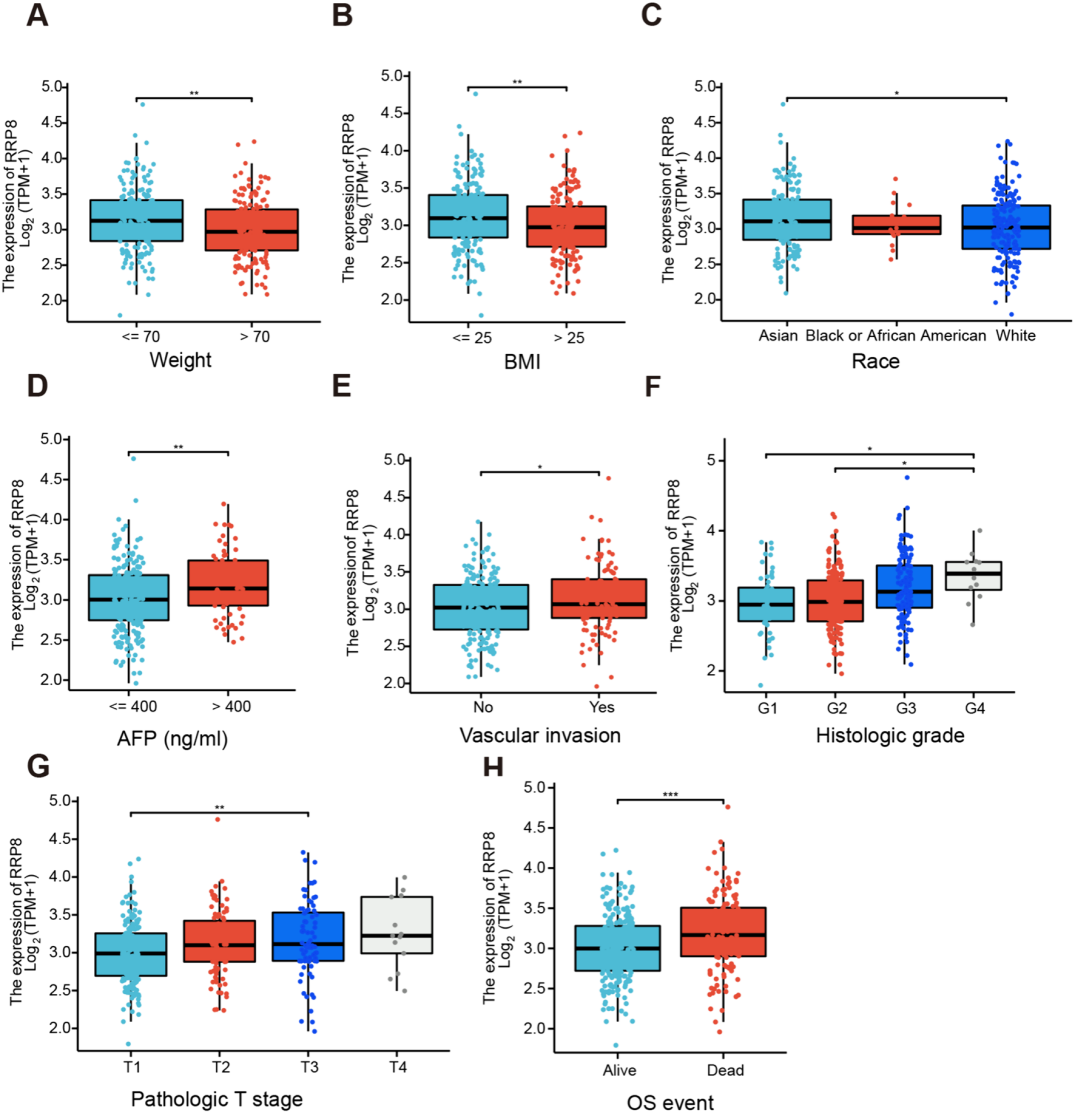
The relationship between RRP8 expression and clinicopathological features. Expression levels of RRP8 were analyzed in categorized groups of Weight (**A**), BMI (**B**), Race (**C**), AFP (**D**), Vascular invasion (**E**), Histologic grade (**F**), Pathological T stage (**G**), and OS event (**H**) using HCC data from the TCGA database. *BMI* Body Mass Index, *AFP* alpha fetal protein, *OS* Overall Survival; *P < 0.05; **P < 0.01; ***P < 0.001

To explore the clinical features with varying RRP8 expression levels, we accessed HCC information from the TCGA database. Patients without sufficient data were excluded, and 374 HCC patients were ultimately included in further analyses.The results of comprehensive analysis of the relationships between clinical parameters and RRP8 expression are summarized in Table 1. Correlation analysis between RRP8 expression and clinicopathologic variables by utilizing logistic regression are summarized in Supplemental Table 2. Body weight (Fig. 5A), body mass index (BMI) (Fig. 5B), race (Fig. 5C), alpha fetoprotein (AFP) level (Fig. 5D), vascular invasion status (Fig. 5E), histological grade (Fig. 5F), pathological T stage (Fig. 5G), and overall survival (OS) (Fig. 5H) were strongly associated with RRP8 expression. In particular, RRP8 was more highly expressed in the underweight (weight ≤ 70 kg) subgroup than in the overweight subgroup (weight > 70 kg). Statistically significant differences were also observed in the BMI, race, AFP level, histological grade, and pathological T stage subgroup analyses. Furthermore, we revealed that high RRP8 expression was positively associated with vascular invasion, and that was negatively associated with OS. These data suggest that HCC patients with high RRP8 expression appear to have poor prognoses.

**Table 1:** Demographic and clinicopathological parameters in high and low RRP8 expressed patients with hepatocellular carcinoma in TCGA

Characteristics	Low expression of RRP8	High expression of RRP8	*P* value
N	187	187	
Pathologic T stage, n (%)			0.045
T1	104 (28%)	79 (21.3%)	
T2	45 (12.1%)	50 (13.5%)	
T3	33 (8.9%)	47 (12.7%)	
T4	4 (1.1%)	9 (2.4%)	
Pathologic N stage, n (%)			0.716
N0	119 (46.1%)	135 (52.3%)	
N1	1 (0.4%)	3 (1.2%)	
Pathologic M stage, n (%)			0.721
M0	125 (46%)	143 (52.6%)	
M1	1 (0.4%)	3 (1.1%)	
Pathologic stage, n (%)			0.086
Stage I	96 (27.4%)	77 (22%)	
Stage II	42 (12%)	45 (12.9%)	
Stage III	33 (9.4%)	52 (14.9%)	
Stage IV	2 (0.6%)	3 (0.9%)	
Gender, n (%)			0.439
Female	57 (15.2%)	64 (17.1%)	
Male	130 (34.8%)	123 (32.9%)	
Race, n (%)			0.278
Asian	72 (19.9%)	88 (24.3%)	
Black or African American	9 (2.5%)	8 (2.2%)	
White	99 (27.3%)	86 (23.8%)	
Age, n (%)			0.570
≤ 60	86 (23.1%)	91 (24.4%)	
> 60	101 (27.1%)	95 (25.5%)	
BMI, n (%)			0.071
≤ 25	81 (24%)	96 (28.5%)	
> 25	89 (26.4%)	71 (21.1%)	
Residual tumor, n (%)			0.177
R0	168 (48.7%)	159 (46.1%)	
R1	6 (1.7%)	11 (3.2%)	
R2	0 (0%)	1 (0.3%)	
Histologic grade, n (%)			< 0.001
G1	34 (9.2%)	21 (5.7%)	
G2	102 (27.6%)	76 (20.6%)	
G3	48 (13%)	76 (20.6%)	
G4	2 (0.5%)	10 (2.7%)	
AFP (ng/ml), n (%)			0.079
≤ 400	116 (41.4%)	99 (35.4%)	
> 400	27 (9.6%)	38 (13.6%)	
Vascular invasion, n (%)			0.425
No	110 (34.6%)	98 (30.8%)	
Yes	53 (16.7%)	57 (17.9%)	
OS event, n (%)			0.002
Alive	136 (36.4%)	108 (28.9%)	
Dead	51 (13.6%)	79 (21.1%)

**Table 2:** Interacting chemicals of RRP8 from CTD

Category	Chemical name (ID)	Interaction	Category	Chemical name (ID)	Interaction
Heterocyclic compounds	2,3,7,8-tetrachlorodibenzofuran (C014211)	Increases expression	Inorganic chemicals	Nanotubes, Carbon (D037742)	Increases expression
Heterocyclic compounds	Abrine (C496492)	Increases expression	Inorganic chemicals	Oxygen (D010100)	Affects expression
Chemical actions and uses	Air Pollutants (D000393)	Increases expression	Inorganic chemicals	Ozone (D010126)	Increases expression
Organic chemicals	Antimycin A (D000968)	Increases expression	Organic chemicals	Pentachlorophenol (D010416)	Increases expression
Organic chemicals	aristolochic acid I (C000228)	Increases expression	Organic chemicals	Phosphinothricin (C003121)	Increases expression
Organic chemicals	bisphenol A (C006780)	Decreases expression	Complex mixtures	Plant Extracts (D010936)	Increases expression
Organic chemicals	Carbon Tetrachloride (D002251)	Increases expression	Inorganic chemicals	potassium chromate (VI) (C027373)	Increases expression
Heterocyclic compounds	Catechin (D002392)	Decreases expression	Organic chemicals	Pregnenolone Carbonitrile (D011285)	Increases expression
Polycyclic compounds	Cyclosporine (D016572)	Increases expression	Organic chemicals	Resveratrol (D000077185)	Increases expression
Heterocyclic compounds	Deguelin (C107676)	Increases expression	Organic chemicals	schizandrin B (C015499)	Increases expression
Polycyclic compounds	Dexamethasone (D003907)	Affects expression	Complex mixtures	Smoke (D012906)	Decreases expression
Organic chemicals	Dibutyl Phthalate (D003993)	Increases expression	Inorganic chemicals	sodium bichromate (C016104)	Increases expression
Organic chemicals	Dronabinol (D013759)	Increases expression	Inorganic chemicals	Sodium Selenite (D018038)	Increases expression
Organic chemicals	Endosulfan (D004726)	Increases expression	Complex mixtures	Soot (D053260)	Increases expression
Heterocyclic compounds	epigallocatechin gallate (C045651)	Increases expression	Organic chemicals	Temozolomide (D000077204)	Increases expression
Organic chemicals	Ethanol (D000431)	Affects expression	Organic chemicals	Tetrachlorodibenzodioxin (D013749)	Affects expression
Polycyclic compounds	Ethinyl Estradiol (D004997)	Decreases expression	Organic chemicals	Tetradecanoylphorbol Acetate (D013755)	Increases expression
Organic chemicals	Formaldehyde (D005557)	Increases expression	Organic chemicals	Thioacetamide (D013853)	Increases expression
Carbohydrates	Gentamicins (D005839)	Decreases expression	Organic chemicals	Valproic Acid (D014635)	Affects expression
Heterocyclic compounds	Grape Seed Proanthocyanidins (C511402)	Decreases expression	Heterocyclic compounds	Vinclozolin (C025643)	Increases expression
Organic chemicals	Hexabromocyclododecane (C089796)	Increases expression	Inorganic chemicals	Nanotubes, Carbon (D037742)	Increases expression
Heterocyclic compounds	ICG 001 (C492448)	Increases expression	Inorganic chemicals	Oxygen (D010100)	Affects expression
Lipids	Ionomycin (D015759)	Increases expression	Inorganic chemicals	Ozone (D010126)	Increases expression
Heterocyclic compounds	Leflunomide (D000077339)	Increases expression	Organic chemicals	Pentachlorophenol (D010416)	Increases expression
Organic chemicals	Methyl Methanesulfonate (D008741)	Increases expression

### Analysis of the prognostic value of RRP8 in HCC

**Fig. 6:**
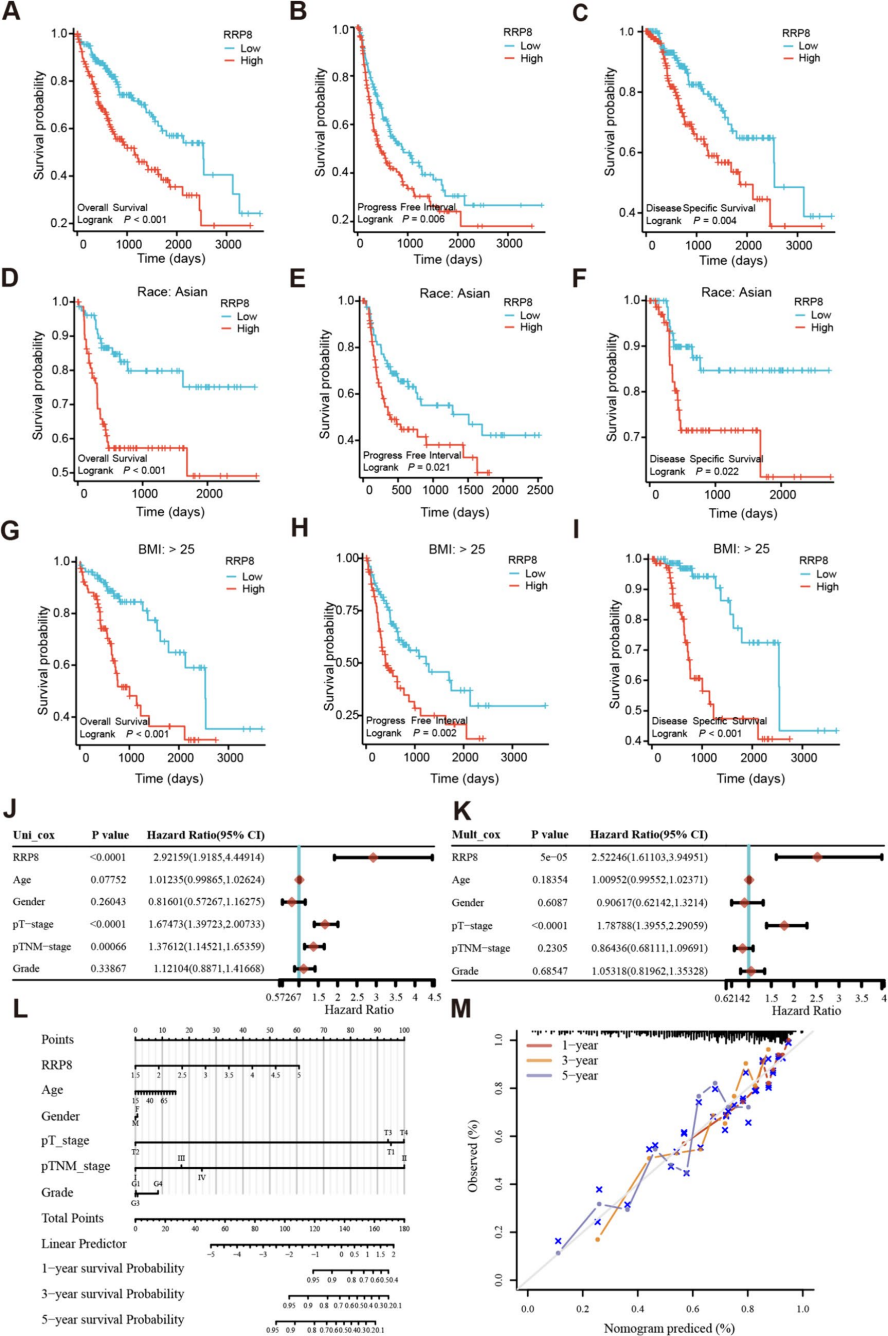
The prognostic analysis of RRP8 in HCC. **A–C** Survival curves of HCC patients with high and low RRP8 expression in terms of OS, PFI and DSS. **D–F** Survival curves of Asian HCC patients with high and low RRP8 expression in terms of OS, PFI, and DSS. **G–I** Survival curves of HCC patients with BMI > 25 divided into high and low RRP8 expression groups in terms of OS, PFI, and DSS. **J** Univariate and **K** multivariate OS prognostic analysis in various patient subgroups based on age, gender, pT-stage, pTNM-stage, grade, and RRP8 expression level. **L** For HCC patients, the nomogram was plotted to estimate OS at the first, third, and fifth year. **M** Nomogram calibration plotted to determine the probability of OS at the first, third, and fifth year. The data comes from the TCGA database. *OS* Overall Survival, *PFI* Progression Free Interval, *DSS* Disease Specific Survival, *pT-stage* pathological Tumor stage, *pTNM-stage* pathological Tumor Node Metastasis stage

Next, we revealed associations between RRP8 expression and OS (Fig. 6A), progression-free interval (PFI) (Fig. 6B), and disease-specific survival (DSS) (Fig. 6C) for HCC patients in TCGA database. High RRP8 expression was negatively correlated with prognosis. Moreover, survival analysis of patients with high RRP8 expression via GEPIA (http://gepia.cancer-pku.cn/) also revealed unfavourable OS and DFS in these patients (Supplementary Fig. 3M–N). Subgroup analysis of Asian patients with low RRP8 expression revealed more favourable OS (Fig. 6D), PFI (Fig. 6E), and DSS (Fig. 6F) outcomes. A subgroup of patients with a BMI > 25 and high RRP8 expression had unfavourable OS (Fig. 6G), PFI (Fig. 6H), and DSS (Fig. 6I) outcomes. Furthermore, HCC patients younger than 60 years with high RRP8 expression had unfavourable OS, PFI, and DSS outcomes (Supplementary Fig. 3A–C). Elevated RRP8 expression with unfavourable OS, PFI, and DSS across all histological grades (Supplementary Fig. 3D–F). Patients with high RRP8 expression and pathological M0 (Supplementary Fig. 3G–I) or N0 (Supplementary Fig. 3J–L) stage disease had poor OS, PFI and DSS.

Univariate and multivariate Cox regression analyses were employed to determine the prognostic factors (Fig. 6J). The multivariate Cox regression analysis results indicated that both pT stage and RRP8 expression were significant variables, suggesting that they are independent factors for OS in HCC patients (Fig. 6K). Based on multivariate Cox regression analysis, a prognostic nomogram incorporating RRP8 expression and other independent clinicopathological factors was developed. Each variable was assigned a score according to the nomogram scale, which was then used to predict patient prognosis at 1, 3, and 5 years. The C-index of the nomogram was determined to be 0.694 (95% CI 0.644–0.743), indicating its good discriminatory power for outcome prediction (Fig. 6L). Furthermore, calibration analysis demonstrated that the predicted values closely aligned with the observed values, further supporting the reliability of our predictions (Fig. 6M). These results align with the multivariate Cox regression analysis.

### Interaction analysis of RRP8 with chemicals

Next, we aimed to discover drugs affecting RRP8 expression via the Comparative Toxicogenomics Database (CTD; https://ctdbase.org/). Data analysis revealed that 49 chemicals may interact with RRP8 and regulate its expression; 37 of these could upregulate RRP8, while 6 could downregulate it. Intriguingly, 6 chemicals with unknown functions could affect RRP8 expression. The 49 chemicals that can regulate the expression of RRP8 include the following main types: heterocyclic compounds, organic chemicals, polycyclic compounds, inorganic chemicals, and complex mixtures (Table 2). In addition, the top 20 connections among RRP8 and other genes were identified using chemical interactions. These findings suggested that RRP8 is closely linked to RNA Polymerase I Subunit E (POLR1E), WD Repeat Domain 43 (WDR43), F-Box Protein FBL12 (FBXL12), NECAP Endocytosis Associated 2 (NECAP2) and COX I Translational Activator (TACO1) (Table 3).

**Table 3:** Relationship of RRP8 with genes via chemical interaction, based on the CTD database

Gene	Similarity index	Common interacting chemicals
POLR1E	0.358024691	29
WDR43	0.336538462	35
FBXL12	0.333333333	24
NECAP2	0.328767123	24
TACO1	0.328125	21
MRPL16	0.324675325	25
NOC4L	0.324324324	24
EMD	0.320987654	26
KNOP1	0.320512821	25
MAK16	0.32	24
KANSL2	0.30952381	26
NOB1	0.30952381	26
PLEKHM2	0.306666667	23
TSR1	0.305882353	26
CCDC86	0.300970874	31
RBM15B	0.296296296	24
WDR77	0.291666667	28
SCRIB	0.290697674	25
MRTO4	0.290322581	27
SLC35F5	0.2875	23

### Functional validation of RRP8 in HCC by two different gene editing systems

**Fig. 7:**
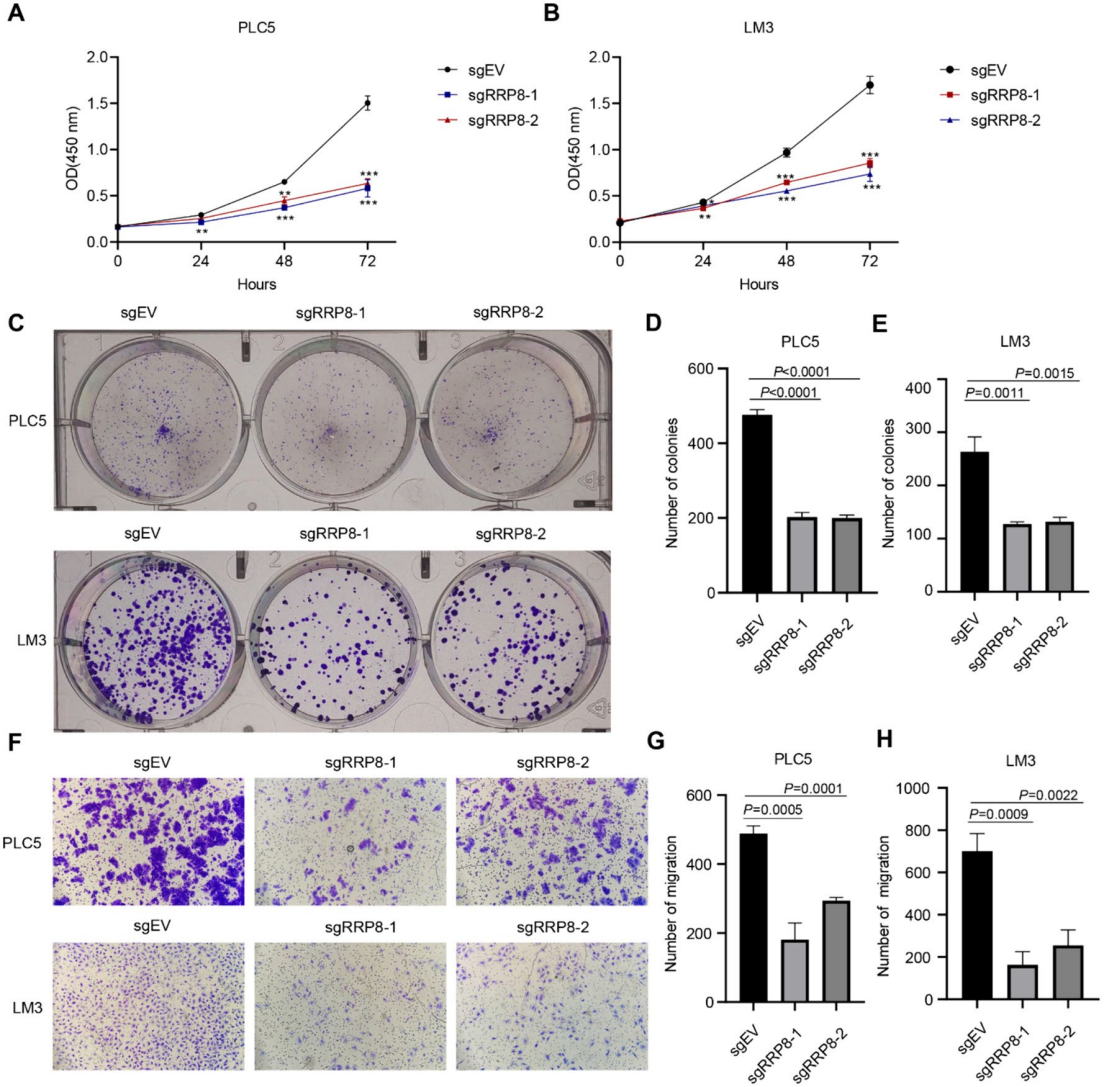
RRP8 is essential for HCC cell proliferation and migration in vitro. **A**, **B** Cell proliferation capacity was examined utilizing CCK-8 assays after RRP8 knockout in PLC5 and LM3 cell lines. **C** Colony formation assay in PLC5 and LM3 cell lines following RRP8 knockout. **D**, **E** Quantative analysis of colony formation assay in PLC5 and LM3 cell lines upon RRP8 knockout. **F** Representative images of transwell assay in PLC5 and LM3 cell lines after RRP8 knockout. **G**, **H** Quantative analysis of migration assay in PLC5 and LM3 cell lines when RRP8 was knockout. EV: empty vector

**Fig. 8:**
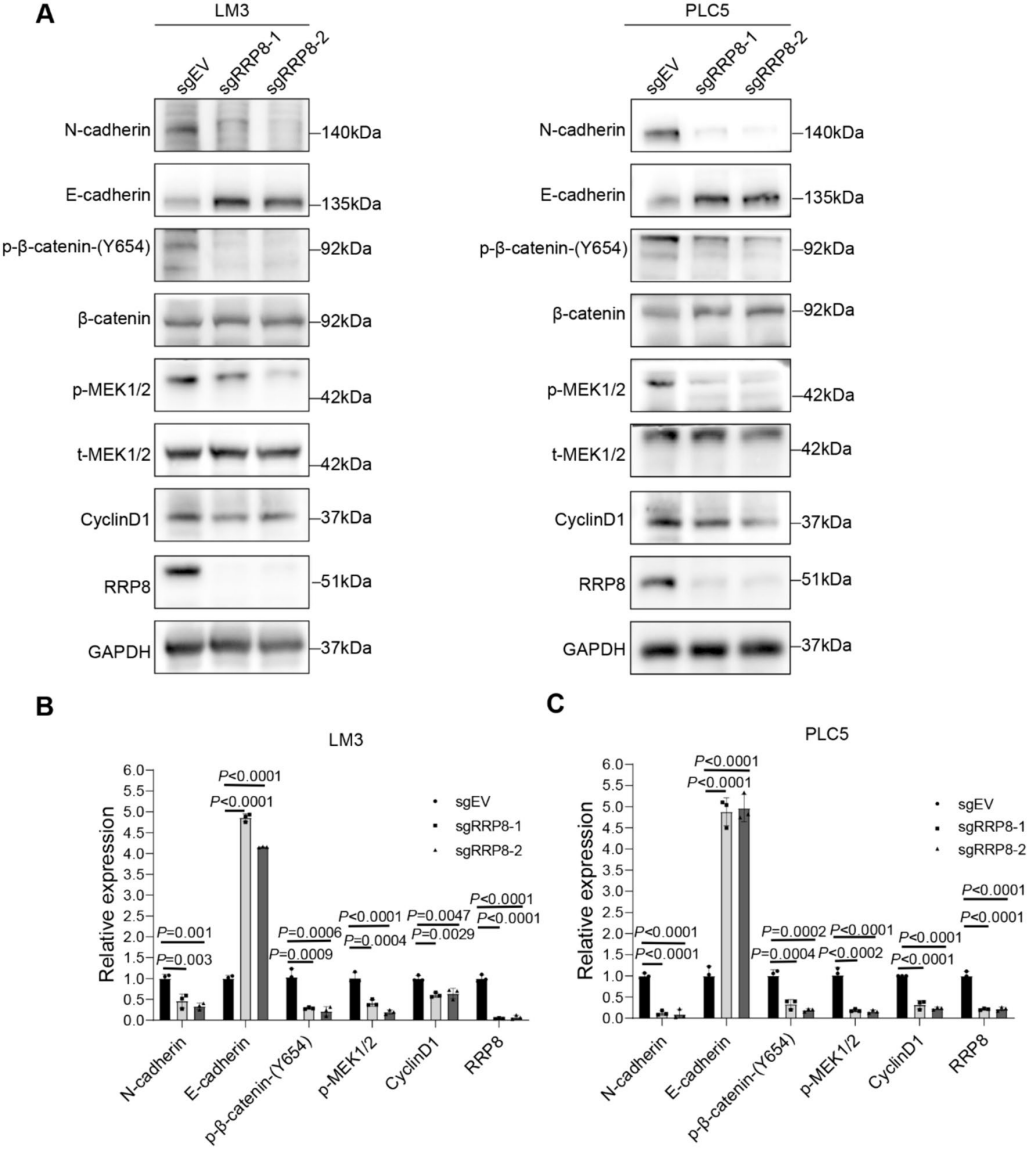
The expressions of MEK1/2, β-catenin and downstream signalling effectors in HCC cells after RRP8 knockout. **A** The expressions of phosphorylation of MEK1/2 and β-catenin-(Y654) were significantly decreased in the RRP8 knockout group compared with the Control group, loss of RRP8 significantly decreased the expressions of some downstream signalling effectors, including cyclin D1, N-cadherin, and increased the expressions of E-cadherin in RRP8 knockout group. **B**, **C** Relative density ratio of RRP8, p-MEK1/2, MEK1/2, p-β-catenin-(Y654), β-catenin, cyclin D1, N-cadherin, E-cadherin protein levels in LM3 and PLC5 cell lines

To further confirm the function of RRP8 in liver cancer, we quantified cell growth after RRP8 knockdown and knockout in PLC5 and LM3 cell lines using doxycycline-inducible shRNAs and single-guide RNAs (sgRNAs), respectively. The RRP8 knockdown efficiency was confirmed at both the transcriptional (Supplementary Fig. 4A–B) and translational (Supplementary Fig. 4C) levels, and the RRP8 knockout efficiency was confirmed at the translational level (Fig. 8A). Loss of RRP8 inhibited the proliferation of PLC5 (Fig. 7A, Supplementary Fig. 4D) and LM3 (Fig. 7B, Supplementary Fig. 4E) liver cancer cells, according to the CCK8 assay. Colony formation assays confirmed that knockdown or knockout of RRP8 consistently impaired colony formation by PLC5 (Fig. 7C, D; Supplementary Fig. 4F, H) and LM3 (Fig. 7C, E; Supplementary Fig. 4G, I) HCC cells. Transwell assays further confirmed that migration was weakened when RRP8 expression was lost in PLC5 (Fig. 7F, G; Supplementary Fig. 4J, L) and LM3 (Fig. 7F, H; Supplementary Fig. 4K, M) cells.

#### Knockout of RRP8 suppressed the activity of the MAPK and β-catenin signalling pathways

To elucidate the underlying mechanisms by which RRP8 suppresses the proliferation and migration of liver cancer cells, the impact of RRP8 expression on several signalling pathways in PLC5 and LM3 cells was examined. Loss of RRP8 expression significantly reduced the phosphorylation of MEK1/2 and β-catenin-(Y654) (Fig. 8A), consistent with the results of GSEA in HCC showing enrichment of the DEGs upregulated by high RRP8 expression in the Wnt and MAPK signalling pathways. Examination of the levels of various downstream signalling effectors revealed that in the RRP8 knockout group, Cyclin D1 and N-cadherin expression was downregulated but E-cadherin expression was upregulated compared to that in the control group (Fig. 8A–C).

## Discussion

In all kingdoms of life, RNAs undergo post-transcriptional modification. RRP8 was discovered to be able to carry out N1-methyladenosine (m1A) modification in yeast 25S rRNA and mammalian 28S rRNA (Peifer et al. 2013; Yokoyama et al. 2018), particularly m(1)A645 alteration of 25S rRNA (Waku et al. 2016). The RRP8 complex promotes the establishment of rDNA heterochromatin while suppressing rRNA transcription. The absence of nucleolar transcription suppression could lead to tumour recurrence following chemotherapy (Yang et al. 2015). RRP8 is also required for the formation of eNoSC, a protein complex that senses the energy state and can control rRNA transcription (Yang et al. 2023). It was reported that RRP8 binds to H3K9me2 and interacts with SIRT1 and SUV39H1, which are necessary for energy-dependent transcriptional suppression (Murayama et al. 2008). However, the roles and pathological functions of RRP8 in cancer, especially those in HCC, have yet to be determined.

In this study, we first verified that the RRP8 mRNA and protein levels were significantly elevated in HCC tissues compared to normal tissues, in line with results from the TCGA and HPA databases. We further confirmed that RRP8 was highly expressed in HCC cell lines. Additionally, based on the ROC curve analysis results, we found that RRP8 could serve as an attractive predictive biomarker to distinguish tumour tissues from normal tissues or peritumoral tissues. Furthermore, we constructed PPI networks based on the DEGs using the Cytoscape platform. ESR1, GRM5, EGF, GAST, AFP, SOX2, LAMC2, GRIA2, GRIN2A and GRM1 were the top 10 hub genes examined, and five core gene clusters (with a total score ≥ 5000) were examined. Recently, many studies have reported that ESR1, EGF, GAST, AFP, SOX2, and LAMC2 are involved in HCC progression. ESR1 inhibited HCC cell proliferation and improved HCC patient prognosis by interacting with the HS1BP3 promoter (Hu et al. 2022). HOXD3 can promote invasion, metastasis, and angiogenesis in HCC by targeting the promoter region of EGF through the EGFR-ERK1/2 signalling pathway (Wang et al. 2021). GAST was identified as a promising prognostic indicator of survival outcomes in individuals with HCC (Hong et al. 2021). Deletion of AFP in HepG2 cells inhibited cell proliferation through inactivation of the PI3K/AKT signalling pathway (Xu et al. 2023). SOX2 is a stem cell transcription factor that controls self-renewal and pluripotency in cancer stem cells, and upregulation of SOX2 could be used as a marker for evaluating prognosis and treatment effectiveness for HCC (Hosseini-khah et al. 2021). LAMC2, encoding the laminin subunit gamma 2 protein, emerged as the most prominent hub gene exhibiting increased involvement in the progression of HCC (Hu et al. 2020).

Tumour-infiltrating immune cells (TIICs), which have recently received increasing attention, play a crucial regulatory role in tumour development (Tian et al. 2020). Research has demonstrated that BRCA1-associated protein (BRAP) is a crucial protein involved in promoting tumour progression through its role in tumour immunity (Ju et al. 2020). Additionally, its correlation with poor prognosis in HCC may be influenced by the presence of TIICs. These TIICs contribute to a complex network of cellular interactions within the tumour microenvironment, leading to an immunosuppressive environment that facilitates immune evasion and ultimately supports tumour growth. Alterations in the immune milieu of the liver can also result in the development of liver lesions, including chronic inflammation and fibrosis/cirrhosis, which are common pathological conditions observed in the liver (Li et al. 2016; Ruf et al. 2020). Furthermore, TIICs play a critical role in shaping the tumour microenvironment (TME) (Liu et al. 2023). Emerging evidence suggests that locoregional treatments for HCC can induce changes within the TME, including increased expression of growth factors, release of tumour antigens, infiltration of cytotoxic lymphocytes, and modulation of both adaptive and innate immune responses (da Fonseca and Araujo 2022).

Our data revealed the closely connection between immune cell infiltration and RRP8 expression. RRP8 expression was closely linked to Th17, DC, NK CD56bright and Th2 cell infiltration in liver cancer. Th17 cells are effector CD4^+^ T cells that contribute to immunological functions as well as autoinflammatory diseases (Martin-Orozco et al. 2009; Peng et al. 2021; Yang et al. 2020). Th17 cells have the capacity to differentiate into two distinct cell subsets, Th1 and Th2, which aid in maintaining the immune response to internal and external pathogens (Bettelli et al. 2006). Th1/17 cells secrete IFN-γ, which stimulates anticancer immune responses (Chatterjee et al. 2018). Previous studies have shown that increased Th17 cell infiltration inhibits breast cancer and lung cancer cell growth (Chang et al. 2014; Karpisheh et al. 2022). DCs are pivotal in the initiation and regulation of immune responses, and DCs have been shown to have anticancer effects (Martinek et al. 2019). Our findings suggest that high RRP8 expression may promote an alteration in the Th2/Th1 balance and favour a switch towards a Th2 response, which is vital for HCC metastasis (Budhu and Wang 2006). One subset of T helper cells called Th2 cells can suppress the host immune response, thereby facilitating the progression of malignancy (DeNardo et al. 2009). IL-4 produced by Th2 cells has been shown to activate a number of cancer-related pathways (Dey et al. 2020; Zhao et al. 2015). Based on prior studies, we could conclude that high RRP8 expression could stimulate immune infiltration during liver carcinogenesis and development. The relationships between RRP8 expression and clinical characteristics were assessed through both Cox univariate and multivariate regression analyses. Multivariate regression analysis suggested that RRP8 may serve as a potential independent risk factor in hepatocellular carcinoma.

The results of GSEA indicated that increased RRP8 expression is linked to the stimulation of DNA replication and cell cycle regulation. These findings imply that RRP8 may play a regulatory role in the growth of tumour cells in liver cancer. Our study revealed that the inhibition of RRP8 by either genetic knockdown or knockout significantly impaired the proliferation and migration of PLC5 and LM3 cells. This study also revealed a close relationship between the elevated expression of RRP8 and the activation of the MAPK and WNT/β-catenin signalling pathways, as determined by GSEA.

MEK1/2 serve as crucial components of the mitogen-activated protein kinase (MAPK) pathway. Multiple studies have indicated a correlation between the activation of the MEK1/2 signalling axis and the progression of human malignancies (Rahima 2019; Tangudu et al. 2019). Recent research indicated that the Wnt/β-catenin signalling pathway is implicated in the advancement of breast cancer through Mortalin-induced EMT (Zhang et al. 2021). Additionally, overexpression of TTYH3 in CCA cells was found to induce EMT, as evidenced by changes in the expression levels of E-cadherin, N-cadherin, and vimentin. This process also involved alterations in the P-GSK3β and β-catenin levels (Xue et al. 2021). The WNT/β-catenin signalling pathway is pivotal in the pathogenesis of liver cancer, influencing various cellular processes, such as proliferation, migration, survival, and epithelial–mesenchymal transition (EMT) (Lou et al. 2023). It is widely recognized that the E-cadherin and N-cadherin proteins are crucial regulatory proteins throughout the EMT process. Additionally, Cyclin D1 is known to play critical roles in various cellular processes associated with liver cancer.

In the present study, we determined that RRP8 knockout downregulated the phosphorylation of MEK1/2 and β-catenin-(Y654) and their downstream effectors, including Cyclin D1 and N-cadherin, and upregulated the level of E-cadherin in PLC5 and LM3 cells. It has been postulated that the activation of the MAPK and β-catenin signalling pathways may play a pivotal role in the progression of RRP8-overexpressing tumours, suggesting that RRP8 may be a potential target for curing HCC.

Taken together, although we performed an integrative analysis of RRP8 and validated its function in HCC, our research has several limitations. First, the mRNA level of RRP8 and the main regulatory networks related to RRP8 in HCC should be verified and investigated through multicentre analysis of specimens. Second, additional molecular evidence and in vivo assays are required for elucidation of the functional roles of RRP8 in HCC.

## Conclusions

In this research, we carried out a comprehensive investigation of RRP8 and explored its pathological functions in HCC. The novel finding indicated that RRP8 was overexpressed in liver cancer specimens and that RRP8 overexpression was correlated with an unfavourable prognosis. Furthermore, RRP8 expression was linked to various immune cells and may influence HCC immune infiltration. Finally, loss of RRP8 expression inhibited liver cancer cells to proliferate and to migrate in vitro. Further research indicated that RRP8 might increase the malignancy of liver cancer by activating the MAPK and β-catenin pathways. In conclusion, these data demonstrated that RRP8 could be an attractive biomarker and therapeutic target for HCC.

## Supplementary Information

Below is the link to the electronic supplementary material.

Supplementary file1 (DOCX 3027 KB)

Supplementary file2 (XLSX 10 KB)

Supplementary file3 (DOCX 20 KB)

Supplementary file4 (XLSX 6917 KB)

Supplementary file5 (DOCX 16 KB)

Supplementary file6 (XLSX 55 KB)

Supplementary file7 (XLSX 178 KB)

Supplementary file8 (XLSX 40 KB)

## Data Availability

The study analyzed datasets that are accessible to the public. These datasets are available at the following location: The repository/repositories' names and accession number(s) can be found in the article/ Supplementary Materials.
