# Supplementary material for: RRP8, associated with immune infiltration, is a prospective therapeutic target in hepatocellular carcinoma
Source: J Cancer Res Clin Oncol. 2024 May 9;150(5):245. doi: 10.1007/s00432-024-05756-9 (PMC11082032; doi:10.1007/s00432-024-05756-9)
Supplement: Supplementary file 1 — Supplementary file1 (DOCX 3027 KB) [file 432_2024_5756_MOESM1_ESM.docx]

**Supplementary Materials**

## Supplementary Figures


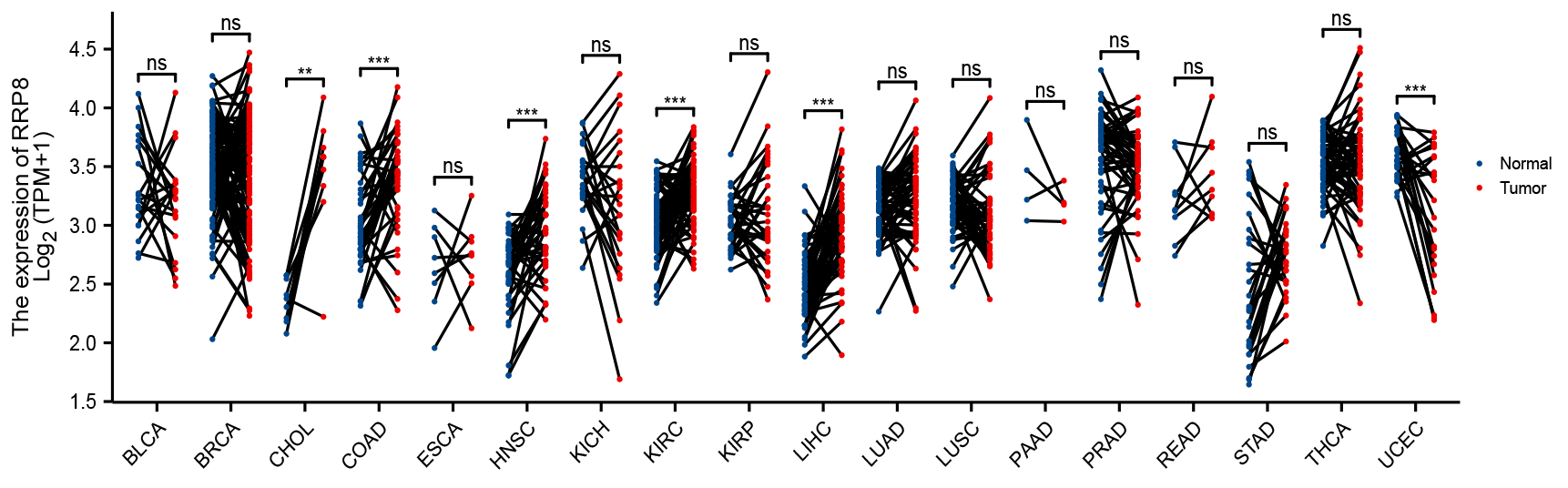


**Supplementary Fig. 1** Comparison of RRP8 expression between tumor and paired normal samples (TCGA). **P*< 0.05, ***P*< 0.01, ****P*< 0.001; ns: no significant differences


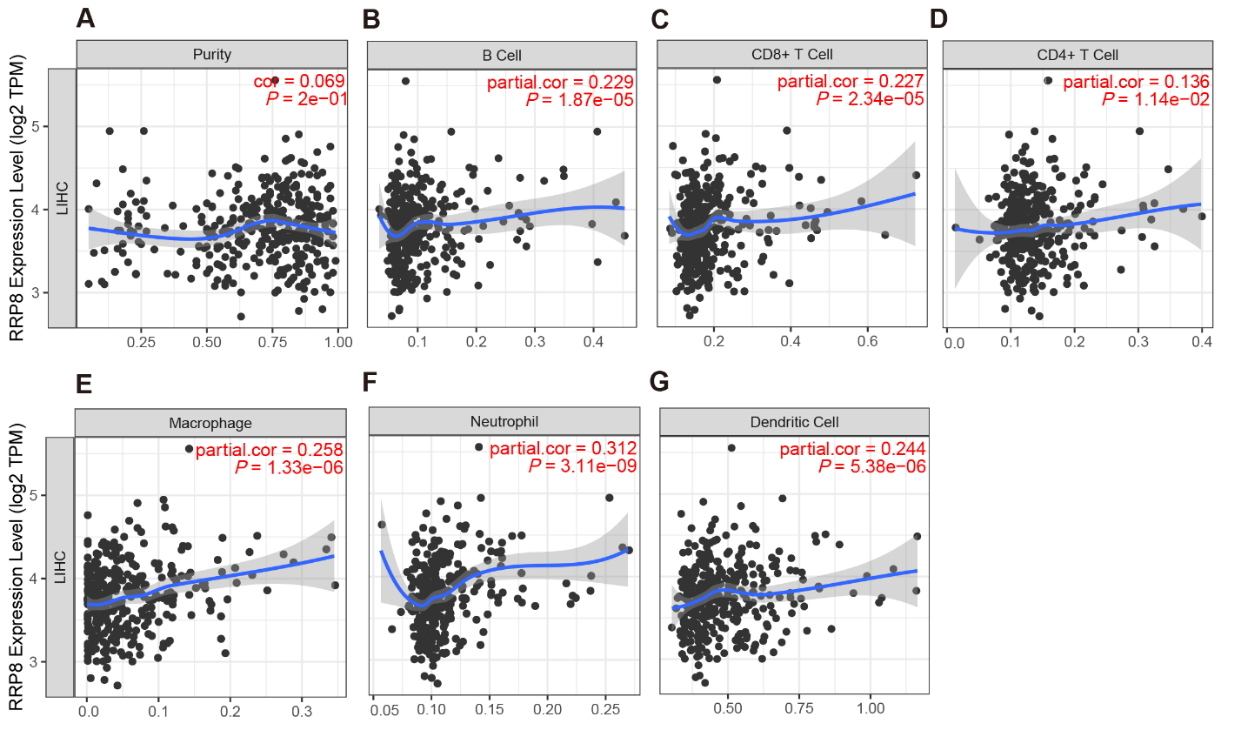


**Supplementary Fig. 2** The correlation between RRP8 expression and immune cell infiltration in HCC (TIMER). After adjustment based on tumor purity **(A)**, RRP8 was positively related to B cell **(B)**, CD8^+^ T cell **(C)**, CD4^+^T cell **(D)**, Macrophage **(E)**, Neutrophil **(F)** and Dendritic cell **(G)** in LIHC. LIHC: Liver hepatocellular carcinoma; cor: correlation


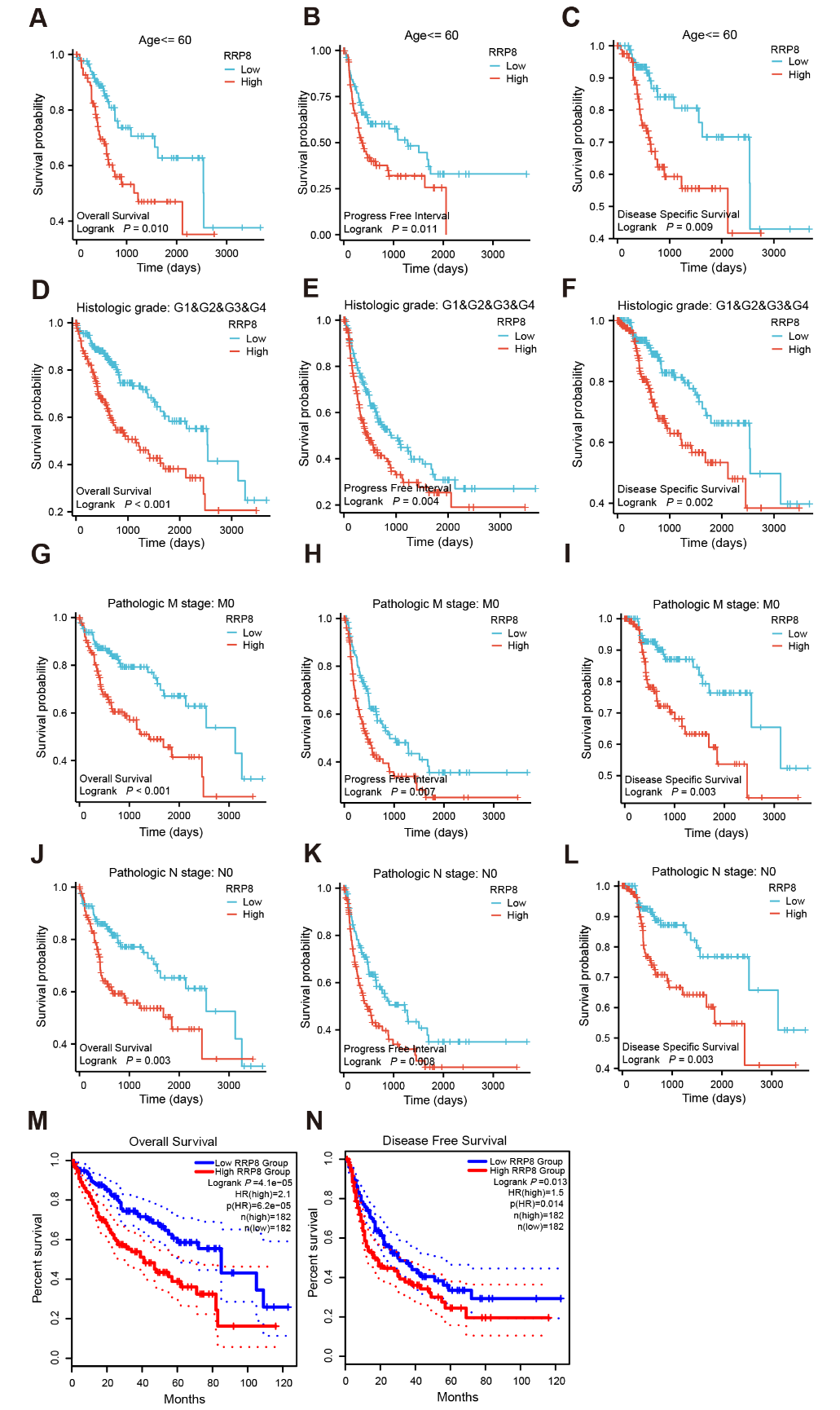


**Supplementary Fig. 3** Analysis of the prognostic values of RRP8 in HCC. **(A–C)** Survival curves of OS, PFI and DSS for patients with age≤ 60. **(D–F)** Graphs of OS, PFI and DSS for HCC patients in all histologic grades with varying levels of RRP8 expression. **(G–I)** Graphical representations of OS, PFI and DSS for patients with HCC in M0 stage. **(J–L)** Graphs of OS, PFI and DSS for patients with HCC in N0 stage characterized by high or low RRP8 expression. **(A–L)** The data comes from the TCGA database. **(M–N)** OS and DFS graphs for HCC patients with low and high RRP8 expression obtained from GEPIA database. OS: Overall Survival; PFI: Progression Free Interval; DSS: Disease Specific Survival


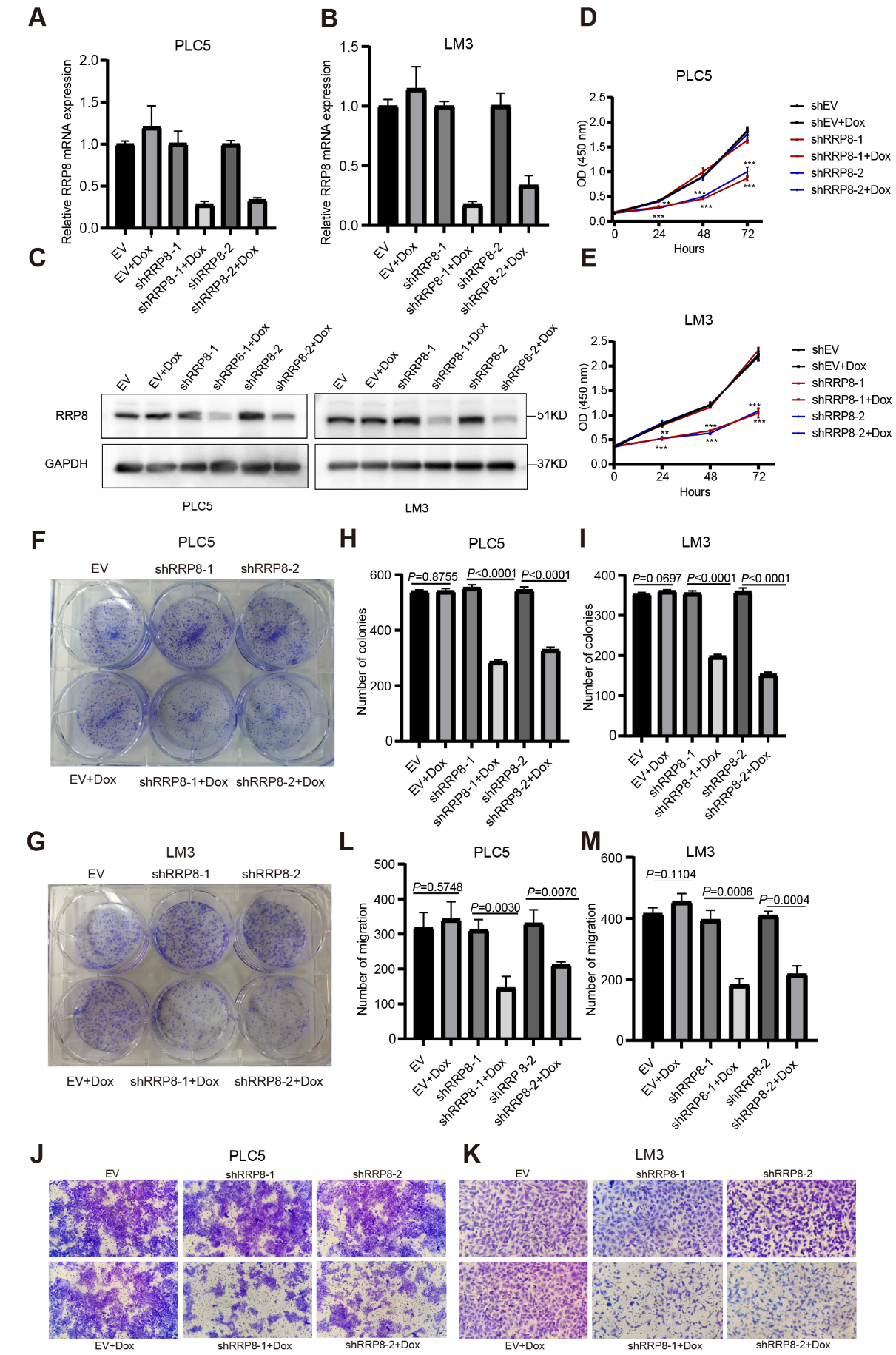


**Supplementary Fig. 4** **RRP8 is essential for HCC cell proliferation and migration in vitro.** **(A–B)** Knockdown efficiency of RRP8 in PLC5 and LM3 cell lines was evaluated utilizing qRT-PCR. **(C)** Knockdown of RPP8 was confirmed by Western blotting at the protein level in PLC5 and LM3 cell lines. **(D–E)** Cell proliferation capacity was examined utilizing CCK-8 assays after RRP8 knockdown in PLC5 and LM3 cell lines. (**F–G**) Colony formation assay in PLC5 and LM3 cell lines following RRP8 knockdown. **(H–I)** Quantitative analysis of colony formation assay in PLC5 and LM3 cell lines upon RRP8 knockdown. **(J–K)** Representative images of transwell assay in PLC5 and LM3 cell lines after RRP8 knockdown. **(L–M)** Quantitative analysis of migration assay in PLC5 and LM3 cell lines when RRP8 was knockdown. EV: empty vector; Dox: doxycycline

## Supplementary Tables

**Supplementary Table 1**. Characteristics of HCC patients from TCGA

**Supplementary Table 2**. Correlation analysis between RRP8 expression and clinicopathologic variables by utilizing logistic regression

**Supplementary Table 3**. RRP8-related DEGs

**Supplementary Table 4**. PCR primer sequences, shRNA and sgRNA target oligonucleotides of RRP8

**Supplementary Table 5**. Analysis of the enrichment of RRP8 with GO and KEGG

**Supplementary Table 6**. GSEA enriched analysis of RRP8

**Supplementary Table 7**. PPI network of RRP8
