# Supplementary material for: RRP8, associated with immune infiltration, is a prospective therapeutic target in hepatocellular carcinoma
Source: J Cancer Res Clin Oncol. 2024 May 9;150(5):245. doi: 10.1007/s00432-024-05756-9 (PMC11082032; doi:10.1007/s00432-024-05756-9)
Supplement: Supplementary file 3 — Supplementary file3 (DOCX 20 KB) [file 432_2024_5756_MOESM3_ESM.docx]

| **Supplementary Table 2 \|** **Correlation analysis between RRP8 expression and**  **clinicopathologic variables by utilizing logistic regression** | | | |
| --- | --- | --- | --- |
| **Characteristics** | **Total (N)** | **Odds Ratio (OR)** | ***P* value** |
| T stage (T2&T3&T4 vs. T1) | 371 | 1.702 (1.130-2.572) | **0.011** |
| N stage (N1 vs. N0) | 258 | 2.644 (0.334-53.852) | 0.402 |
| M stage (M1 vs. M0) | 272 | 2.622 (0.331-53.382) | 0.406 |
| Pathologic stage (Stage III&Stage IV vs. Stage I&Stage II) | 350 | 1.778 (1.095-2.916) | **0.021** |
| Tumor status (With tumor vs. Tumor free) | 355 | 1.436 (0.943-2.194) | 0.093 |
| Gender (Male vs. Female) | 374 | 0.843 (0.545-1.300) | 0.439 |
| Race (Black or African American&White vs. Asian) | 362 | 0.712 (0.469-1.079) | 0.110 |
| Age (>60 vs. <=60) | 373 | 0.889 (0.591-1.335) | 0.570 |
| Weight (>70 vs. <=70) | 346 | 0.571 (0.372-0.873) | **0.010** |
| BMI (>25 vs. <=25) | 337 | 0.673 (0.437-1.033) | 0.071 |
| Residual tumor (R1&R2 vs. R0) | 345 | 2.113 (0.800-6.194) | 0.144 |
| Histologic grade (G3&G4 vs. G1&G2) | 369 | 2.412 (1.566-3.744) | **<0.001** |
| Vascular invasion (Yes vs. No) | 318 | 1.207 (0.760-1.920) | 0.425 |
| AFP (ng/ml) (>400 vs. <=400) | 280 | 1.649 (0.944-2.913) | 0.081 |
| Child-Pugh grade (B&C vs. A) | 241 | 1.303 (0.540-3.208) | 0.556 |
| Adjacent hepatic tissue inflammation (Mild&Severe vs. None) | 237 | 1.337 (0.802-2.237) | 0.266 |
| Albumin(g/dl) (>=3.5 vs. <3.5) | 300 | 0.883 (0.515-1.514) | 0.651 |
| Fibrosis ishak score (1/2&3/4&5/6 vs. 0) | 215 | 1.079 (0.616-1.898) | 0.790 |
