# Supplementary material for: RRP8, associated with immune infiltration, is a prospective therapeutic target in hepatocellular carcinoma
Source: J Cancer Res Clin Oncol. 2024 May 9;150(5):245. doi: 10.1007/s00432-024-05756-9 (PMC11082032; doi:10.1007/s00432-024-05756-9)
Supplement: Supplementary file 5 — Supplementary file5 (DOCX 16 KB) [file 432_2024_5756_MOESM5_ESM.docx]

**Supplementary Table 4. PCR primer sequences, shRNA and sgRNA target oligonucleotides**

**of RRP8**

| **Gene** | **Direction** | **Sequence（5’-3’）** |
| --- | --- | --- |
| RRP8 | Forward | ATTGTCTCCAAGGACCTGACCAACA |
|  | Reverse | GCTTGTAGAGACATGGCTGAAGCTG |
| GAPDH | Forward | TCTCCTCTGACTTCAACAGCGAC |
|  | Reverse | CCTGTTGCTGTAGCCAAATTCGTTG |

| **shRNA** | **Target** **oligonucleotides** |
| --- | --- |
| shRRP8-1 | GACCAAGCTAGGCTTCAAGAT |
| shRRP8-2 | CCTGACCAACAGCCATTTCTT |

| **sgRNA** | **Target oligonucleotides** |
| --- | --- |
| sgRRP8-1 | ACAGCCAGGGGTTGCTAGAG |
| sgRRP8-2 | GCTCTTCGAACATGAGGGTC |
